# Exercise Stress Testing in Children with Metabolic or Neuromuscular Disorders

**DOI:** 10.1155/2010/254829

**Published:** 2010-07-15

**Authors:** Tim Takken, Wim G. Groen, Erik H. Hulzebos, Cornelia G. Ernsting, Peter M. van Hasselt, Berthil H. Prinsen, Paul J. Helders, Gepke Visser

**Affiliations:** ^1^Child Development and Exercise Center, Wilhelmina Children's Hospital, University Medical Center Utrecht, NL 3508 AB Utrecht, The Netherlands; ^2^Faculty of Medicine, Vrije University Medical Center, NL 1007 MB Amsterdam, The Netherlands; ^3^Department of Metabolic Diseases, Wilhelmina Children's Hospital, University Medical Center Utrecht, NL 3508 AB Utrecht, The Netherlands; ^4^Department of Metabolic and Endocrine Diseases, Wilhelmina Children's Hospital, University Medical Center Utrecht, NL 3508 AB Utrecht, The Netherlands

## Abstract

The role of exercise as a diagnostic or therapeutic tool in patients with a metabolic disease (MD) or neuromuscular disorder (NMD) is relatively underresearched. In this paper we describe the metabolic profiles during exercise in 13 children (9 boys, 4 girls, age 5–15 yrs) with a diagnosed MD or NMD. Graded cardiopulmonary exercise tests and/or a 90-min prolonged submaximal exercise test were performed. During exercise, respiratory gas-exchange and heart rate were monitored; blood and urine samples were collected for biochemical analysis at set time points. Several characteristics in our patient group were observed, which reflected the differences in pathophysiology of the various disorders. Metabolic profiles during exercises CPET and PXT seem helpful in the evaluation of patients with a MD or NMD.

## 1. Introduction

Metabolic diseases (MDs) and Neuromuscular disorders (NMDs) comprise a large heterogeneous group of diseases, that directly (via intrinsic muscle pathology or defective metabolic pathways) or indirectly (via nerve pathology), impair muscle function and result in exercise intolerance.

Although the value of exercise tests in patients with MD/NMD has been acknowledged for several decades [1–3], the role of exercise stress tests as a diagnostic or evaluative tool in children and adults with MD/NMD is relatively underresearched. Moreover, exercise stress tests are not standard for most centers to be performed in clinical care [4–7]. In this paper, we provide two standardized exercise tests with preliminary metabolic profiles in children with a diagnosed MD/NMD for this purpose. 

Exercise stress tests in patients with a metabolic disorder involved in ATP synthesis show a clear specific metabolic profile during exercise [4]. These metabolic profiles can be useful as a reference for identifying patients for a possible MD or NMD. 

Therefore, the aim of the current study was to describe the metabolic profiles during exercise in children with a diagnosed NMD or MD. This information might be helpful for clinicians in the diagnosis and follow-up of patients with these disorders.

## 2. Methods

### 2.1. Subjects

In this retrospective chart review, patients with an established diagnosis involving general ATP synthesis or a dystrophinopathy, who were referred for exercise stress testing to the Departments of Metabolic Diseases and Child Development and Exercise Center, University Medical Center Utrecht, the Netherlands, were included. 

Thirteen patients (9 ♂, 4 ♀, age 5–15 years) with an established diagnoses were studied in detail. Diagnoses were Glycogen Storage Disease (GSD) type 1a (1x), GSD type 3 (1x), GSD type 7 (1x), Medium-Chain Acyl CoA dehydrogenase deficiency (MCAD (2x)), Short-Chain Acyl CoA dehydrogenase deficiency (SCAD (1x)), Multiple Acyl CoA dehydrogenase deficiency (MADD (2x)), ketothiolase deficiency (1x), mitochondrial myopathy (1x), Hypokalemic episodic paralysis (1x), and dystrophinopathy (Becker Muscular Dystrophy (BMD) (2x).

### 2.2. Exercise Tests

Two exercise stress tests, respectively, a cardiopulmonary exercise test (CPET) and a prolonged exercise ergometry test (PXT) were performed following a standardized protocol [8]. Blood samples were taken, immediately before and directly after the CPET and PXT, and analyzed for lactate, creatine kinase (CK), ammonia, acylcarnitines, and organic acid. Urine samples were collected up to three hours after the exercise test and further analyzed for creatinine, organic acid, amino acid, tetraglucose, purine, and pyrimidine [8]. 

A CPET (to determine the peak oxygen uptake [VO_2peak_] and peak workload [W_peak_]) and a submaximal PXT (90 minutes at 30% of W_peak_) were performed in the morning. After a light breakfast, the patients performed a symptom-limited CPET on a bicycle ergometer. Workload was increased in constant increments of 10, 15, or 20 watts every minute, depending on the patients' length [9] and was in some conditions adjusted for the physical condition of the patient. This protocol was continued until the patient stopped due to volitional exhaustion, despite strong verbal encouragement. During the tests, all subjects breathed through a facemask (Hans Rudolph Inc., Kansas City, MO), connected to a calibrated respiratory gas analysis system (Oxygen Champion/Pro, Care Fusion, Houten, The Netherlands). This system measured breath-by-breath minute ventilation (VE), oxygen uptake (VO_2_), and respiratory exchange ratio (RER = VCO_2_/VO_2_) using conventional equations. During the maximal exercise test, heart rate (HR) was measured continuously by a bipolar electrocardiogram. Peak HR (HR_peak_), VO_2peak_, VO_2peak_/kg, and peak RER were taken as the average values over the last 30 seconds of the test. 

A PXT was performed one week after the maximal exercise test to prevent interference from the previous test. The PXT consisted of a 90-minute cycling at a constant work rate of 30%  W_peak_, as described previously [8]. 

### 2.3. Blood and Urine Sampling and Analysis

Blood samples were obtained from an indwelling catheter inserted into a vein of the dorsum of the hand. Five milliliters of blood from each sample was placed in lithium-heparin tube, except for determination of FFA and ammonia; respectively, normal blood (without Li-heparin) and EDTA (another anticoagulant) blood was used for the analysis [8]. After taking the blood, the tubes were stored in ice and brought to the laboratory. 

Blood taken before and after the CPET was analyzed for lactate, creatine kinase (CK), ammonia, acylcarnitines, and organic acid. Urine samples taken before and after the CPET, were analyzed for creatinine, organic acid, amino acid, and tetraglucose, until three hours after the exercise test. 

During the PXT, blood samples were taken at regular time intervals (*t* = 0, 30, 60, 75, 90, 105, and 120 minutes after the start of the exercise). Concentrations of glucose, lactate, CK, free fatty acids (FFA), ammonia, 3-OH-butyric acid and 3-keto-butyric acid, acylcarnitines, and organic acid were determined. Before and up to three hours after exercise, urine samples were obtained and analyzed for creatinine, organic acid, amino acid, and tetraglucose. 

Glucose, lactate, CK, and ammonia were determined with a Beckman Coulter DxC chemical analysis machine (Fullerton, USA). Enzymatic method was used for determination of FFA, 3-ketobutyric acid, and 3-OH-butyric acid. After lipoprotein lipase hydrolyzed triglyceride into fat acids and glycerol, free glycerol was measured colorimetrically.

Organic acid concentration in urine and plasma was determined by gas chromatography-mass spectrometry as their trimethylsilyl derivates (Hewlett Packard 5890 series II gas chromatograph linked to a HP 5989B MS-Engine mass spectrometer (Hewlett Packard, Avondale, PA)). The coefficients of variation for the various measured organic acids varied between 10%–15%. Analysis of acylcarnitine in plasma as their butyl esters was performed by electrospray tandem mass spectrometry (ESI-MS-MS; Micromass Quattro Ultima, Micromass Ltd., UK) equipped with an Alliance HPLC system (Waters, Milford, MA, USA). Also for these analyses the coefficients of variation for the determined acylcarnitines were 10%–15%. Analysis of amino acids in plasma and urine was done with amino acid analyzer (ion-exchange chromatography-ninhydrin).

## 3. Results

### 3.1. CPET

As expected, patients with a MD/NMD showed abnormal results on the CPET (Tables 1 and 2). Patient 2 (GSD-3) stopped the CPET because of myalgia in the lower limbs, compared to reference values for healthy children [10, 11], the patients with GSD-3, MCAD, SCAD, and mitochondrial myopathy (patients 4, 6, and 10, resp.) had a significantly reduced HR_peak_. RER_peak_ was significantly lower in the patient with GSD-3 (patient 2) and also in the patient with MCAD (patient 4) and surprisingly increased to 1.0 in the patient with GSD-7 (patient 3). VO_2peak_ and VO_2peak_/kg were significantly lower in the patients 3, 4, 10, and 13. These were patients with GSD-7, MCAD, mitochondrial myopathy, and Hypokalemic episodic paralysis, respectively. 

A remarkably high VO_2peak_/kg was observed in one of the patients with BMD (patient 12).

The patients with GSD-1a and mitochondrial myopathy (patient 1 and 10, resp.) had significantly increased lactate concentrations at rest. Patient 2, with GSD-3, had an increased CK values at rest and after exercise. The 2 patients with BMD (patients 11 and 12) showed persistently highly elevated CK levels. One patient (patient 13) showed mildly elevated CK.

### 3.2. PXT

Biochemical profiles of the MD/NMD patients during the PXT varied with the disorder (Table 3). Two patients, one with GSD-1a and the other with mitochondrial myopathy (resp., patient 1 and 10), showed significantly increased concentrations of blood lactate at all time points. The patient, with GSD-7 had significantly increased ammonia concentrations with no rise in lactate during exercise. 

During and after exercise, the CK value of the patient with GSD-7 (patient 3) was significantly increased as well as in the 2 patients with BMD (patients 11 and 12). 

Acylcarnitines C6, C8, C10, C12, and C14:1 were all increased in two patients with MADD (patients 7 and 8) in rest as well as during exercise. The patient with ketothiolase deficiency (patient 9) had increased C5:1 and C5-OH acylcarnitine during rest and exercise, as well as several increased organic acids in the urine. In the patient with mitochondrial myopathy (patient 10), C5 carnitine was increased in the urine during and after exercise. In the patient with SCAD (patient 6), there was no C4 carnitine found. In all other MD/NMD patients, no altered acylcarnitines, carnitines, for organic acids concentrations could be observed in plasma or urine (data not shown). 

## 4. Discussion

The purpose of this study was to describe metabolic profiles during exercise using CPET and PXT including extensive blood and urine analyses in children with a diagnosed MD/NMD. This information might be helpful for clinicians in the diagnosis and follow-up of these disorders. Because of the heterogeneity of the disorders, there was a large variation in the CPET and PXT results between patients. These differences reflect the different pathophysiology of the various disorders (e.g., defects in different metabolic pathways) and heterogeneity within disorders. 

Metabolic profiling might be helpful in the further workup towards a diagnosis. For example, a low rise in lactate after CPET is suggestive for a GSD, and a very high increase in lactate, combined with a very low VO_2peak_, might be suggestive for a mitochondrial myopathy. Further studies should develop an algorithm for the interpretation of exercise data in MD/NMD patients, comparable to the interpretative algorithms for cardiac and pulmonary limitations during exercise [12, 13].

The diagnostic yield of exercise stress testing in children with unexplained exercise intolerance seems relatively low. Among 29 patients referred for exercise intolerance of unknown origin, only 3 patients could be diagnosed with a MD/NMD: 2 patients with a Becker Muscular Dystrophinopathy and one patient with a hypokalemic episodic paralysis. However, many of these patients have undergone extensive medical screening before they were referred for exercise testing. Ten percent is therefore a reasonable yield. It is our opinion that the expense of exercise testing including extensive blood and urine analyses is justified because it could be useful for guiding the diagnostic workup and can differentiate between patients with medically unexplained exercise intolerance and patients with a MD/NMD. In patients with a MD involved in ATP synthesis, only during certain periods of metabolic stress (e.g., exercise, fasting, or illness), abnormal quantities of metabolites in blood and urine can be found, and symptoms are present. These defects can only be indentified using standardized tests. The current paper provides two standardized exercise tests with preliminary metabolic profiles for this purpose.

Furthermore, several of the tested MD/NMD patients (patients 3, 7, 8, and 10) were referred for exercise testing to assess their exercise capacity for physical activity recommendations. Based on their exercise results, an advice regarding appropriate levels of physical activity was provided. Sufficient amounts of physical activity are necessary for an optimal physical, psychosocial, and emotional development in children [14].

In addition, for patient 12, we gave an exercise restriction based on the findings. This patient was a talented cyclist with a very high VO_2peak_ for his age. However, during several races he developed myoglobinuria, and he had quite high resting values of CK. A muscle biopsy in the workup after the tests revealed a duplication in exon 24-29 of the dystrophin gene, and the diagnosis of BMD was made. Based on these results, the boy was advised to stop high-level cycling because of the increased risk of renal failure due to myoglobinuria. 

One of the limitations of this clinical report is the small and heterogeneous population. This reflects the rarity of the disorders. Therefore, multicentred studies are needed to increase the sample size for each of the disorders. Further, it is important that these profiles are established in children as not all metabolic profiles seen in adults are valid in children. For example, a recent study showed that the well-known second-wind phenomenon in patients with McArdle's disease (GSD5), which is considered as a diagnostic feature of this disease [15], was not observed in children with McArdle's disease [16]. 

## 5. Conclusion

In this paper we describe the metabolic profiles during exercise in 13 children with a diagnosed MD/NMD. Metabolic profiles during exercise were of assistance in diagnosing 3 patients with rare presentations of MD/NMDs. In addition, exercise stress testing was helpful for the prescription of appropriate levels of physical activity. 

## Figures and Tables

**Table 1:** Cardiopulmonary measurements of patients during the CPET.

Patient	Diagnosis	Determination of diagnosis	Age (years)	Sex	Weight (kg)	BMI (Z-score)	HR_peak_ (beats/min)	RER_peak_	VO_2peak_ (L/min) (Z-score)	VO_2peak_ /kg (mL/min/kg) (Z-score)
1	GSD-1a	Mutation R570X en delta F327	11.9	M	40	19.3 (0.88)	205	1.16	1.99 (−0.04)	49.7 (−0.42)
2	GSD-III	Debranching enzyme deficiency in leucocytes	11.9	F	39	19.6 (0.71)	182	0.92*	1.45 (−1.5)	37.1 (−1.5)
3	GSD-7	Phosphofructokinase deficiency in muscle	12.9	M	32	13.1 (−3.7)	184	1.0	1.78 (−3.55)	34.6 (−3.24)
4	MCAD	MCAD deficiency in leucocytes homozygous Lys329Glu mutation	5.4	F	28	19.4 (1.98)	134	0.92*	0.55 (−3.5)	19.5 (−4.2)
5	MCAD	MCAD deficiency in leucocytes	11.4	F	42	18.9 (0.59)	NA	NA	NA	NA
6	SCAD	SCAD deficiency in leucocytes and fibroblasts; mutation	7.0	M	25	14.6 (0.70)	173	1.05	1.00 (−0.86)	40.1 (−1.9)
7	MADD	MADD deficiency in fibroblasts	10.2	M	33	16.1 (−0.23)	195	1.25	1.33 (−1.1)	40.4 (−1.8)
8	MADD	MADD deficiency in fibroblasts	8.6	M	25	14.1 (−1.35)	180	1.21	1.29 (0.09)	51.5 (−0.26)
9	2-Methylacetoacetyl-CoA-thiolase deficiency	2-Methylacetoacetyl-CoA-thiolase deficiency in fibroblasts	8.7	M	30	15.5 (−0.32)	179	1.20	1.30 (−0.69)	43.5 (−1.4)
10	Mitochondrial respiratory chain myopathy	Diminished ATP production in fresh muscle biopsy	9.7	M	34	16.9 (0.30)	152	1.39	0.63 (−3.6)	18.5 (−5.0)
11	M. Becker dystrophinopathy	Duplication exon 24-29 dystrophin gene	10.4	M	25	12.4 (−3.6)	NA	NA	NA	NA
12	M. Becker dystrophinopathy	Duplication exon 24-29 dystrophin gene	14.8	M	57	18.6 (−0.22)	202	1.26	1.57 (1.7)	62.6 (2.7)
13	Hypokalemic episodic paralysis	Arg1239His mutation in CACNA1S-gene	13.8	F	62	24.5 (1.63)	218	1.25	1.20 (−2.0)	19.4 (−4.0)

*Abbreviations*: BMI: Body Mass Index, HR_peak_: peak heart rate, VO_2peak_: peak O_2_ uptake, RER_peak_: peak respiratory exchange ratio, *: significantly different from normal, NA: not assessed.

**Table 2:** Biochemical measurements of patients before and after the CPET.

Patient	Lactate		CK		Ammonia	
	(mmol/L)		(U/L)		(mmol/L)	
	Before	After	Before	After	Before	After
1	3.5*	10.3	NA	NA	5	4*
2	2.2	1.6	540*	597*	NA	NA
3	0.8	2.4	246*	273*	61*	337*
7	1.3	7.3	125	146	NA	NA
8	1.3	6.0	79	89	NA	NA
9	1.5	4.3	116	121	17	12
10	3.1*	6.9	85	100	8	6*
12	1.5	13*	577*	773*	34*	54
Normal values Mean (range)	1.56 (0.7–2.3)	7.0 (3.2–11.4)	104 (45–192)	123 (51–234)	18 (9–23)	42 (10–94)

*Legend*: NA: not assessed, *: significantly different from normal.

**Table 3:** Biochemical measurements of patients during the PXT test.

Subject	Time (min)	Glucose (mmol/L)	Lactate (mmol/L)	FFA (mmol/L)	3-Keto-B (mmol/L)	3-OH-B (mmol/L)	CK (U/L)	Ammonia (*μ*mol/L)
1	0	4.9	4.3*	0.51	0.14	0.0	116	
	30	4.4	4.6*	0.52	0.13	0.02	115	
	60	5.7*	2.8*	0.489	0.12	0.02	119	
	75	5.6	3.1*	0.536	0.12	0.02	119	
	90	6.7*	2.9*	0.559	0.12	0.02	121	
	15 after	7.4*	4.1*	0.792	0.13	0.04	121	
	30 after	7.5*	4.9*	0.666	0.12	0.04	118	

3	0	5.5	1.8	0.08	0.0	0.0	166	22
	30	5.9	1.2	0.14	0.0	0.0	181*	
	60	5.8*	0.8	0.23	0.0	0.0	186*	
	75	6.9*	1.2	0.36	0.09	0.04	187	275*
	15 after	6.9	2.3	0.25	0.0	0.03	183*	
	30 after		2.0	0.26	0.0	0.0	184*	144*

6	0	4.6	3.2*	0.268	0.12	0.0	48	
	30	4.6	1.3	0.302	0.1	0.0	46	
	60	4.4	1.4	0.444	0.12	0.0	48	
	15 after	4.6	1.1	0.503	0.13	0.03		
	30 after	4.7	0.9	0.528	0.13	0.03	51	

7	0	7.1	1.1	0.76	0.07	0.09	103	
	30	5.6	1.2	0.28	0.0	0.0		
	60	4.9	1.0	0.49	0.0	0.0	98	
	75	4.8	1.3	0.88	0.05	0.05	105	
	90	5.5	1.1	1.03	0.10	0.12	119	
	30 after	4.8	1.0	0.93	0.15	0.26	117	

8	0	5.2	2.0	0.15	0.0	0.0	58	
	30	4.6	0.9	0.31	0.0	0.0		
	60	4.3	0.9	0.62	0.07	0.04	80	
	75	4.4	1.0	0.91			78	
	30 after	4.5	1.2	1.34	0.14	0.24	78	

9	0	6.4	1.4	0.22	0.09	0.03	90	14
	30	4.9	1.4	0.18	0.0	0.0	93	
	60	4.7	1.3	0.21	0.0	0.0	89	
	75	4.4	1.1	0.42	0.0	0.0	99	22
	90	4.9	1.1	0.64	0.11	0.11	91	
	15 after	4.8	0.8	0.69	0.13	0.18	92	
	30 after	4.6	1.0	0.55	0.14	0.2	92	14

10	0	4.0	2.4*	0.20	0.11	0.10*	138	20
	30	3.7*	8.6*	0.21	0.11	0.11*	156	
	60	3.5*	9.6*	0.31	0.14	0.13	151	
	75	3.6*	9.5*	0.49	0.16	0.16	156	20
	90	3.6*	9.7*	0.71	0.16	0.18	157	
	15 after	4.1	7.0*	0.72	0.113	0.29	145	
	30 after	4.0	4.7*	0.73	0.18	0.29	146	

11	0	5.9	1.4	0.41	0.0	0.0	5020*	7.0
	30	4.8	1.4	0.19	0.0	0.0	4975*	
	15 after	5.3	1.4	0.57	0.0	0.0	5036*	
	30 after	5.2	1.3	0.5	0.0	0.0		18
12	0	5.1	1.1	0.15	0.0	0.0	695*	33*
	30	5.3	1.2	0.08	0.0	0.0	776*	
	60	5	1.2	0.11	0.0	0.0	774*	
	75	5.1	1.4	0.16*	0.0	0.0	771*	51*
	90	5.0	1.7	0.23	0.0	0.0	766*	
	15 after	5.1	1.1	0.76	0.0	0.0	755*	

*Legend*: FFA: free fatty acids, 3-keto-B: 3-ketobutaric acid, 3-OH-B: 3-hydroxybutaric acid, *: significantly different from normal.
